# Exploring the use of deep learning models for accurate tracking of 3D zebrafish trajectories

**DOI:** 10.3389/fbioe.2024.1461264

**Published:** 2024-09-25

**Authors:** Yi-Ling Fan, Ching-Han Hsu, Fang-Rong Hsu, Lun-De Liao

**Affiliations:** ^1^ Institute of Biomedical Engineering and Nanomedicine, National Health Research Institutes, Miaoli, Taiwan; ^2^ Department of Biomedical Engineering and Environmental Sciences, National Tsing-Hua University, Hsinchu, Taiwan; ^3^ Department of Information Engineering and Computer Science, Feng Chia University, Taichung, Taiwan

**Keywords:** bioengineering, zebrafish, trajectory tracking, object recognition, translational

## Abstract

Zebrafish are ideal model organisms for various fields of biological research, including genetics, neural transmission patterns, disease and drug testing, and heart disease studies, because of their unique ability to regenerate cardiac muscle. Tracking zebrafish trajectories is essential for understanding their behavior, physiological states, and disease associations. While 2D tracking methods are limited, 3D tracking provides more accurate descriptions of their movements, leading to a comprehensive understanding of their behavior. In this study, we used deep learning models to track the 3D movements of zebrafish. Videos were captured by two custom-made cameras, and 21,360 images were labeled for the dataset. The YOLOv7 model was trained using hyperparameter tuning, with the top- and side-view camera models trained using the v7x.pt and v7.pt weights, respectively, over 300 iterations with 10,680 data points each. The models achieved impressive results, with an accuracy of 98.7% and a recall of 98.1% based on the test set. The collected data were also used to generate dynamic 3D trajectories. Based on a test set with 3,632 3D coordinates, the final model detected 173.11% more coordinates than the initial model. Compared to the ground truth, the maximum and minimum errors decreased by 97.39% and 86.36%, respectively, and the average error decreased by 90.5%.This study presents a feasible 3D tracking method for zebrafish trajectories. The results can be used for further analysis of movement-related behavioral data, contributing to experimental research utilizing zebrafish.

## 1 Introduction

Model organisms are crucial in research fields like genetics, molecular biology, and cell biology. They are vital in pharmaceutical development, disease mechanism discovery, and clinical therapy applications. Common model organisms include mice, rabbits, and zebrafish. The zebrafish (*Danio rerio*), a small tropical freshwater fish from Southeast Asia, is ideal for research due to its strong reproductive ability, rapid development, high transparency, simple genome, and ease of experimental manipulation. These traits make zebrafish invaluable in biology, developmental biology, genetics, toxicology, and drug research (Darland and Dowling, 2001; Gerlai et al., 2000; Guo, 2004; Levin et al., 2003; Linney et al., 2004). The zebrafish and human genomes share high homology, with 70% of human genes having an ortholog in zebrafish and 80% of human disease-related genes having equivalents in zebrafish (Braithwaite et al., 1996; Lieschke and Currie, 2007). This homology makes zebrafish key in cancer, cardiovascular, and neurological research, enabling significant progress in understanding disease mechanisms and developing therapies. Animal behavior studies often validate results, a method widely used across various domains.

Understanding the locomotor behaviors and trajectories of animals in different environments is essential for studying their physiological, behavioral, and cognitive aspects. Therefore, tracking zebrafish trajectories is crucial. In zebrafish studies, 3D trajectories are more informative than 2D trajectories due to the complexity of their movements in three-dimensional space. While 2D data can provide some insights, it cannot fully capture the diversity of zebrafish movements (Jouary et al., 2016). 3D trajectories offer a more comprehensive understanding of zebrafish locomotor behavior and movement capabilities. Most studies use 3D methods to accurately reconstruct and analyze zebrafish movement (Maaswinkel et al., 2013; Qian and Chen, 2017; Audira et al., 2018). These methods are beneficial for quantitatively analyzing zebrafish behaviors and habits in different environments, enhancing our understanding of their behavioral and physiological characteristics. Experimental models require accurate, reliable, and repeatable detection of subjects’ spatiotemporal positions (Stuart et al., 1990).

AI can be utilized to track zebrafish trajectories across various applications, including: 1) Behavioral Research–tracking zebrafish movements in response to stimuli such as light and sound, which provides insights into their behavior, social interactions, and sensory processing (Yang et al., 2021; Wang et al., 2018; Zhang et al., 2013; Sun et al., 2019; Haurum et al., 2020; Barreiros et al., 2021; Bashirzade et al., 2022); 2) Drug Discovery and Toxicity Testing–observing zebrafish responses to different drugs to evaluate their efficacy and potential toxicity (Yang et al., 2021; Zhang et al., 2013; Haurum et al., 2020; Bashirzade et al., 2022; Zhang et al., 2021; Bozhko et al., 2022); 3) Environmental Monitoring–monitoring zebrafish activity in controlled environments to assess their health, population dynamics, and detect indicators of environmental degradation (Zhang et al., 2013; Liu et al., 2019).

Table 1 compares several widely used zebrafish tracking methods and their associated equipment and features. Noldus, a Dutch company, offers EthoVision XT, a versatile and flexible video tracking software with various configurations for different experimental needs. However, it is expensive and not specifically designed for zebrafish. In 2005, the French company ViewPoint Behavior Technology introduced software for zebrafish behavioral research, enabling automatic tracking and analysis of movement and behavior. This system includes additional devices and software for various analyses, but it is also costly, which can be a barrier for small to medium-sized laboratories. In 2018, G. Audira developed the Zebrafish 3D swim behavior observation system (Audira et al., 2018), including a fish tank, backboard, mirror, and supplementary lighting. This setup records two perspectives in one video using a mirror. However, it is fixed and cannot accommodate different tank shapes or sizes based on experimental requirements. Zebrafish behavior assessment systems typically require multiple expensive and complex devices. Three-dimensional tracking often needs commercial software or complex programming, multicamera synchronization, and high frame rates (60 and 100 frames/second). These methods are semi-automatic, require human intervention, and are challenging due to the variable and non-normally distributed zebrafish behavior data. Larger sample sizes are often necessary. Existing tracking software is limited and expensive, posing difficulties for smaller laboratories. New methods are needed to address these issues.

**TABLE 1:** A comparison of various widely used zebrafish tracking methods, the associated equipment, and their features.

Software	EthoVision XT	ZebraLab	Gilbert audira	Self-developed 3D visual tracking method
Function				
Dimensions	2D	Mainly 2D; 3D hardware needs to be purchased separately	3D	3D✔
Tanks	Single/multiple	Single/multiple	Single	Single
Limit	Limited to pure white background, different fish tanks and experiments require additional software purchase	Limited to pure white background, different fish tanks and experiments require additional software purchase	Limited to pure white background, fish tank size fixed	Striped or pure white background available✔
Object	Adult	Adult and embryo	Adult	Adult
Manual	Manual identification	Manual identification	Manual identification	Automatic identification✔
Dataset labels	Manual identification	Manual identification	Manual identification	Automatic identification✔

EthoVision XT offers many software and equipment options to meet diverse experimental requirements. Although not specifically designed for zebrafish, the complex interface and numerous options provide flexibility and broad applicability. However, the detailed configuration relies on user settings, and the software can be relatively expensive. ZebraLab, designed for zebrafish behavioral research, provides an automated video tracking system capable of tracking and analyzing zebrafish movement, activity, and behavior. The system also offers additional devices and software for various behavioral analyses and experimental designs. Nevertheless, the basic software, behavior analysis plugins, and hardware devices are priced in the range of hundreds of dollars, potentially posing financial challenges for smaller laboratories. The Zebrafish 3D Swim Behavior Observation Aquarium System, developed in collaboration by Gilbert Audira and colleagues at Chung Yuan Christian University, includes a tank, backdrop, mirror, and supplementary lighting. This system records two perspectives in the same video using a mirror. However, due to its fixed nature, it cannot accommodate different-shaped or sized tanks based on experimental design requirements. In this study, a 3D tracking system was used for single zebrafish tracking within a tank. The proposed method is adaptable to both plain and striped backgrounds, effectively minimizing manual intervention during operation.

In this regard, deep learning techniques are useful for accurately tracking zebrafish motion trajectories (Chang et al., 2024; Alzoubi et al., 2024), enabling precise studies of their movement patterns and behavioral performance (Fan et al., 2023). Compared to manual tracking, computer-based methods offer significant advantages. Manual tracking is time-consuming, labor-intensive, and error-prone, whereas computer-based methods are automated, reducing time and ensuring high accuracy and repeatability. With suitable algorithms and parameters, comprehensive and precise trajectory tracking can be achieved by quantifying animal locomotion features in different environments. Deep learning, a branch of machine learning, models neural networks based on the human brain, using multilayered neural networks to learn complex data representations. Compared to traditional machine learning, deep learning offers superior learning and generalization capabilities, automatically extracting features and reducing manual effort. Convolutional neural networks (CNNs), consisting of convolutional and pooling layers, are commonly used for object detection. The process involves feature extraction, object classification, and bounding box regression. Common object detection methods include R-CNN (Girshick, 2014), Fast R-CNN (Girshick, 2012), Faster R-CNN (Shaoqing Ren et al., 2016), and YOLO (you only look once) (Redmon, 2015). YOLO is an end-to-end object detection method and is among the most widely adopted approaches. YOLOv7, an improvement of YOLOv4 (Bochkovskiy et al., 2020) developed by Chien-Yao Wang and his team, addresses performance issues with large datasets (Wang et al., 2022). YOLOv7 incorporates new technologies and optimization methods, such as the perception domain attention (PA) mechanism, anomaly detection, panoramic image recognition, and multiscale fusion. These enhancements improve model accuracy and operational efficiency, making YOLOv7 a stable and efficient object detection framework.

In this study, we employed two synchronized cameras to capture videos, which were subsequently cropped and annotated to create a dataset. Through experimental testing, we determined the most suitable hyperparameters for training a deep learning model for accurate zebrafish recognition. Subsequently, by merging and proportionally transforming the quadrants from the two cameras, we obtained the 3D coordinates of the zebrafish and thus reproduced the 3D zebrafish movement trajectories. We employed a deep learning model for object detection to identify and track the zebrafish. The reconstructed 3D coordinates were used to generate dynamic visualizations of the zebrafish movements within the aquarium. This method significantly enhances the analysis and study of zebrafish behavior.

## 2 Materials and methods

**FIGURE 1:**
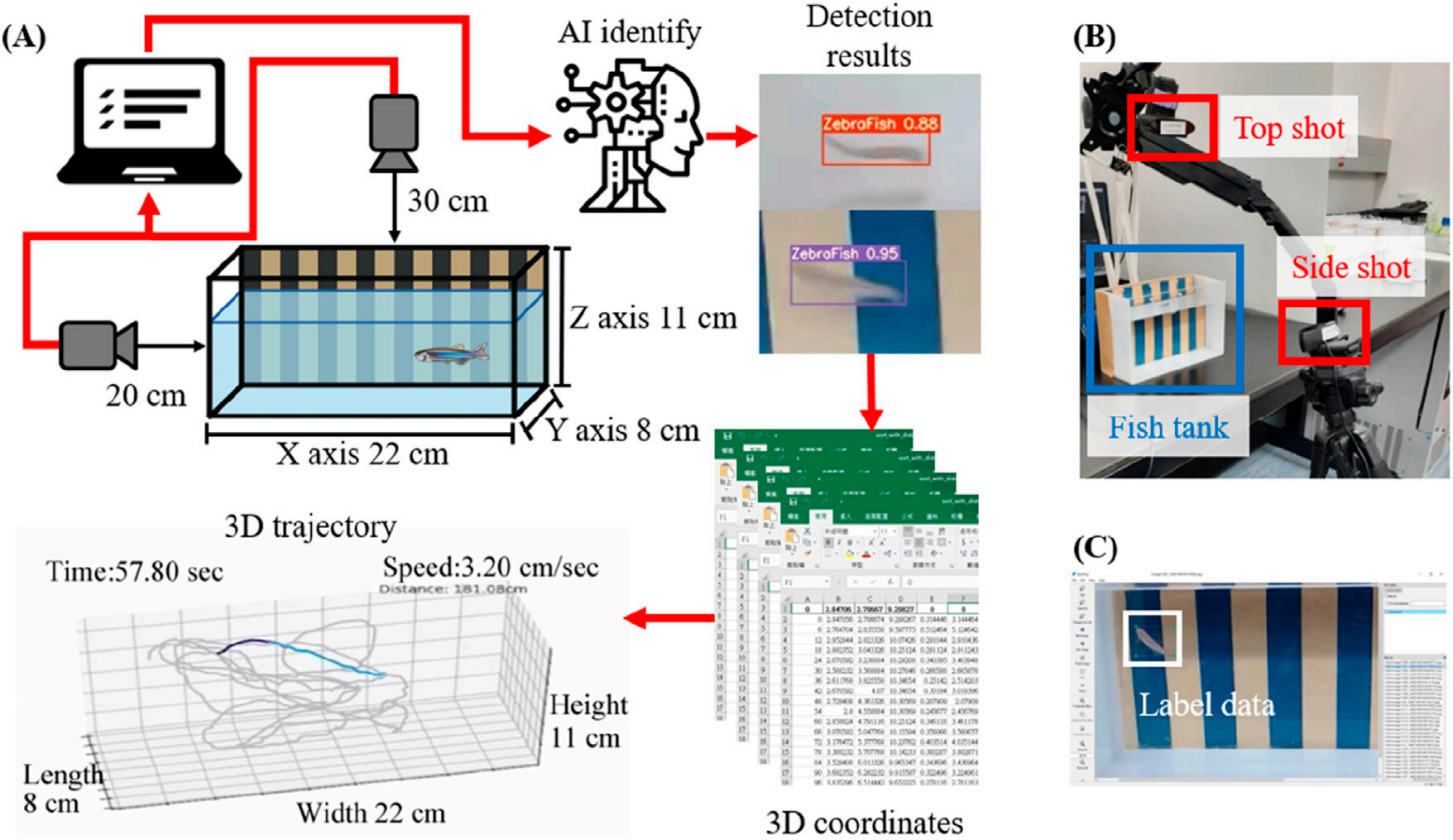
Schematic diagram of the proposed method and the information collection procedure. **(A)** As shown in the schematic diagram, synchronized videos were acquired by simultaneously capturing top-down and side-view perspectives using two cameras. The obtained videos were uniformly cropped, and the images were annotated. Subsequently, a deep learning model was trained using the annotated dataset to determine the position of the zebrafish. By merging and transforming quadrants from two perspectives, 3D coordinates of zebrafish in each frame were obtained. Finally, these coordinate-generated trajectories were connected, and the trajectories were dynamically reconstructed in a 3-axis quadrant diagram, which represented the restored aquarium dimensions. **(B)** The scenario in which two cameras simultaneously capture footage from a top-down and side-view perspective. **(C)** The images were cropped and then labeled using LablImg after processing.

**FIGURE 2:**
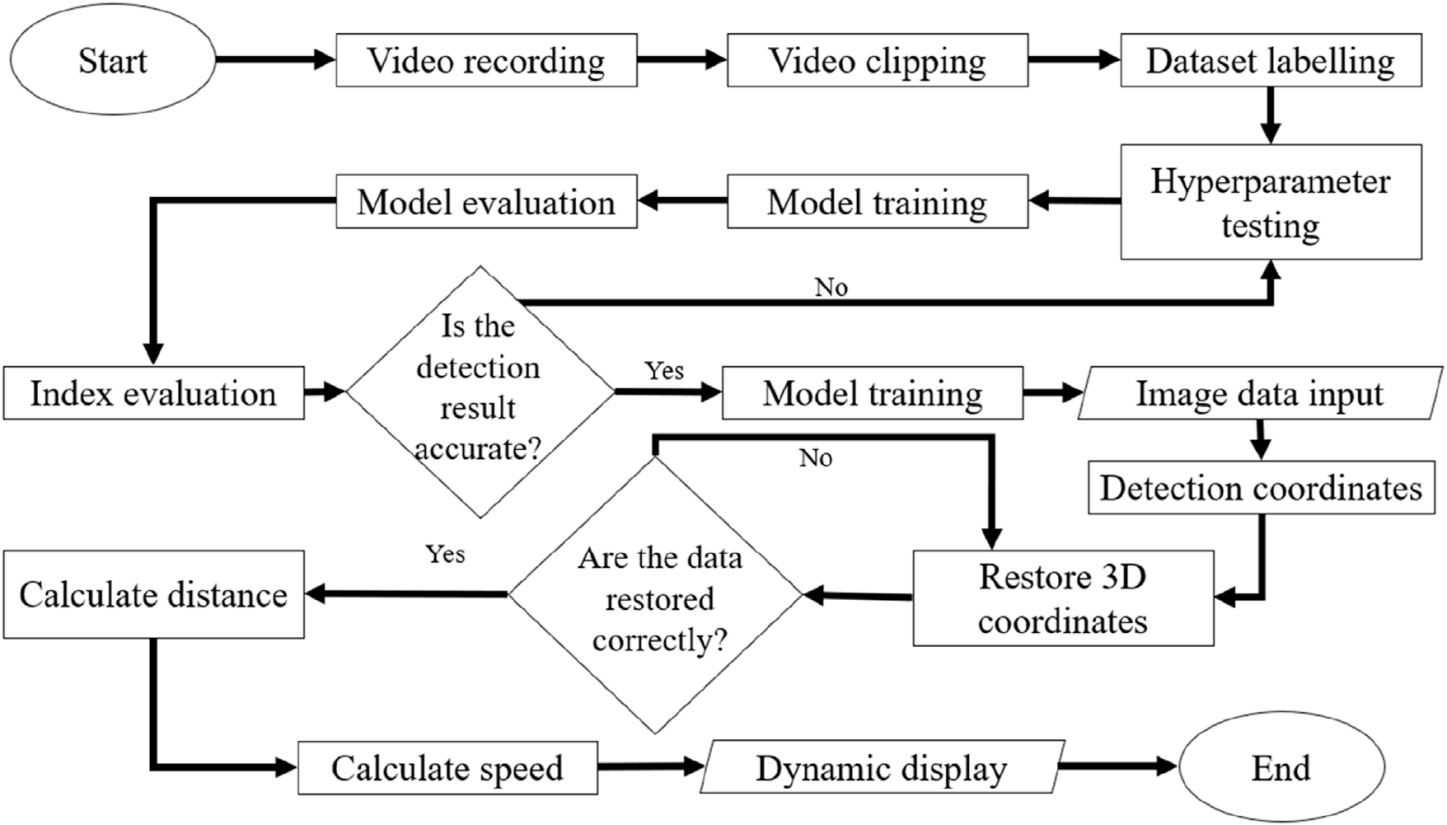
Flow chart of the proposed method. As shown in the procedural diagram of the proposed methodology, two synchronized cameras recorded videos. To transform the footage into an annotatable dataset, the dynamic videos collected by both cameras were segmented into numerous static images. Subsequently, labeling was performed to facilitate recognition by a deep learning model. To achieve optimal model performance and recognition results, preliminary training was conducted using a small dataset with various hyperparameter configurations, and the validation results were compared. With this approach, we identified the most effective parameter settings, which were utilized for model training with the entire dataset. Evaluation metrics were employed to confirm the model’s accuracy. The trained model was employed for the detection and reconstruction of 3D coordinates, facilitating the generation of continuous trajectories. Ultimately, the trajectories were dynamically visualized on a 3-axis quadrant diagram representing the restored aquarium dimensions.

We propose a novel deep learning approach for 3D trajectory tracking, focusing on achieving fully automatic, low-cost detection and visualization of 3D trajectories with minimal human intervention. This method aims to address issues related to trajectory tracking accuracy, continuous tracking, and data representations, providing an improved data analysis approach for zebrafish studies. The proposed framework is illustrated in Figure 1A. Synchronized videos were simultaneously captured by top-down and side-view cameras (Figure 1B). These videos were subsequently cropped and transformed to generate datasets; then, the data were annotated using the labeling software LablImg (Tzutalin, 2017) (Figure 1C). The deep learning model was trained using the annotated data to determine the zebrafish positions. By merging and transforming the quadrants from two perspectives, the 3D coordinates of the zebrafish in each frame were obtained. Initially, a small dataset was used to train models with different hyperparameters to identify the best configuration, which was then used to train the model with the entire dataset. Evaluation metrics confirmed the model’s accuracy. The trained model was then applied for detection and 3D coordinate recognition, followed by trajectory connection. Finally, the trajectories were dynamically displayed (Figure 2).

### 2.1 Data collection and labeling

Two Logitech C922 Pro HD Stream webcams were utilized to capture the required videos at the Taiwan Zebrafish Core Facility, a branch of the National Institute of Health Research in Taiwan. Synchronized recording was performed using two cameras, which captured videos from both the top and side of the fish tank (Figure 1B). A total of 61 min and 346 s were recorded across eight videos, with a frame width and height of 1,920 and 1,080 pixels. A Python program was used to crop one frame every 0.1 s using custom-written Python code, resulting in a total of 36,980 images. Additionally, four videos with a total duration of 2 min were recorded and used as a test set.

To ensure the synchronized start and end of the two camera recordings, the video frames simultaneously displayed the top and side views. Prior to capturing the dataset, we conducted stereo calibration using a calibration board of the same dimensions as the fish tank to ensure the relative positions and orientations of the cameras. After recording, the regions outside the fish tank were cropped to focus on the experimental area. LabelImg software was used to annotate the zebrafish positions within the images, and these annotated images were saved in a format compatible with YOLO for further processing.

We utilized a dataset of 36,980 images and successfully reconstructed the coordinates of 18,490 zebrafish. Zebrafish prefer shallow water areas with high visibility (Lin et al., 2021). Reflections on the water surface can create bright regions below, interfering with visual observations and making tracking beneath the surface challenging. To address this, the dataset was augmented with data from the upper layers of the water column, enhancing the model’s ability to detect zebrafish below the water surface and improving overall detection performance.

**FIGURE 3:**
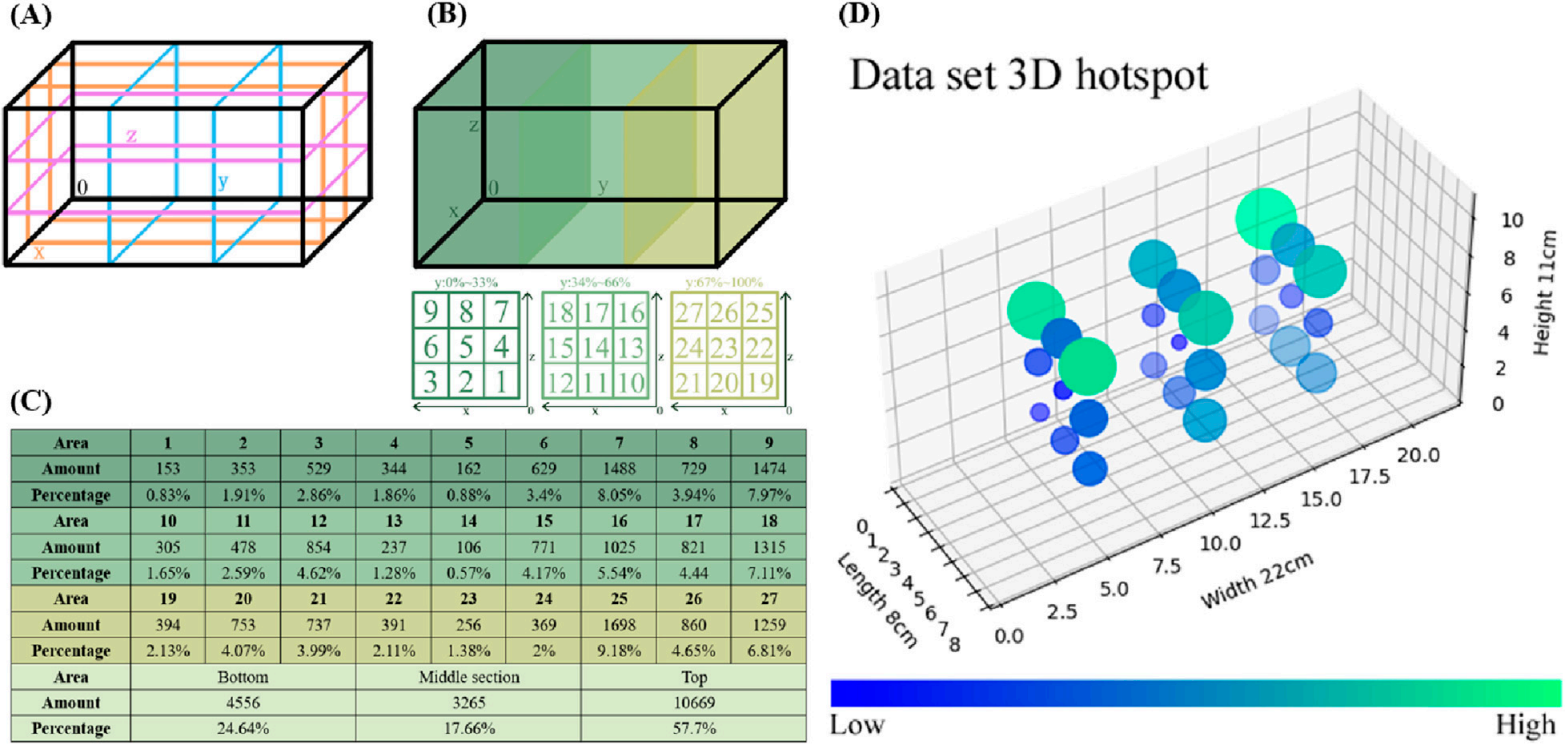
Statistical analysis of the dataset. **(A)** To determine the coordinate distribution of the dataset within the aquarium, the movement range of the zebrafish was divided into three intervals along the X-axis, Y-axis, and Z-axis directions. **(B)** The regions of the 3-axis quadrant are labeled with numerical identifiers, resulting in a total of 27 regions. **(C)** This chart provides a statistical overview of the coordinates within each region, as well as their proportions within the entire dataset. Additionally, the totals and proportions are summarized for the upper, middle, and lower layers. **(D)** For an intuitive representation of the dataset’s distribution, a 3D heatmap was generated to illustrate the spatial pattern of the data. The circle sizes directly reflect the relative proportions, while the color of the circles ranges from dark blue for smaller proportions to bright green for larger proportions.

To represent the data distribution clearly, the zebrafish movement range was divided into three intervals along the X, Y, and Z-axes (Figure 3A). The entire range was further subdivided into 27 regions (Figure 3B), with the number and proportion of zebrafish in each region determined (Figure 3C). The lower, middle, and upper regions included 4,556, 3,265, and 10,669 coordinates, accounting for 24.64%, 16.66%, and 57.7% of the distribution. A 3D heatmap was generated to intuitively represent the dataset’s distribution (Figure 3D). Circle sizes indicate relative proportions, with smaller circles in deep blue and larger circles in bright green.

YOLOv7 is a deep learning model for object detection, introduced with six base models: YOLOv7, YOLOv7x, YOLOv7-d6, YOLOv7-e6, YOLOv7-e6e, and YOLOv7-w6. Each model has a slightly different neural network, and their performance can vary for the same task. To select the optimal weight, we trained all weights and chose the best based on test set performance. In the first round of testing, the top and side-view camera models were trained 300 times using 1,000 annotated images. We compared precision and recall based on these results. In the second round, the camera models were trained 300 times using 3,470 annotated images and were used to detect zebrafish in the same test videos with a 90% confidence level. We evaluated and compared their accuracy, precision, and recall.

We utilized data from two cameras to obtain 3D coordinates through two approaches. The first approach involved merging and annotating images from both cameras to train a unified model, enhancing generalizability. However, mutual interference between images might reduce accuracy. The second approach involved separately annotating images from each camera to obtain distinct models for top- and side-view cameras, improving accuracy but potentially reducing generalizability. To determine the most suitable method, we created two datasets: one with merged images and another with separate images from each camera, each containing 1,000 images. YOLOv7.pt was used as the base model, with 300 training iterations for both datasets. The trained models were then tested with the same test videos at a 70% confidence level, and their accuracy, precision, and recall were compared.

In deep learning, the number of training iterations significantly influences the training outcomes, impacting both model generalizability and overfitting. Therefore, when training deep learning models, different training iterations should be explored to achieve the optimal training results. Using the same dataset, we trained the models with 100, 150, 200, 250, 300, 350, 400, 450, and 500 iterations. Subsequently, each model was tested with the test set, and the accuracy, precision, and recall were compared. The number of training iterations that led to the best performance was selected for the following experiments.

Moreover, deep learning models require a substantial amount of sample data, and the amount of data directly impacts model performance. Insufficient data may result in the model failing to learn enough features, while excessive data might lead to an overly complex model with poor generalizability. Therefore, we explored different dataset sizes and compared the training performance of different models to effectively enhance the performance and accuracy of the proposed deep learning models. We trained the model with the w6 weight for 300 iterations using images collected by the side-view camera as the dataset. Then, models were trained for 3,000, 4,000, 5,000, 6,000, 7,000, 8,000, 9,000, and 10,000 iterations. Subsequently, each model was tested with the test set, and the accuracy, precision, and recall were compared. The dataset that led to the best performance was identified, and this dataset was used in the following experiments.

### 2.2 Trajectory reproduction

After model training, the 3D coordinates were reconstructed based on the detected data. The data collected by both cameras were extracted and restored, and the center of the detection framework was calculated as the coordinate point for display. The X-Y and X-Z axes were separately designated, using the X-axis as the merging criterion. The top camera captures images with the X-Y axes, while the side camera captures images with the X-Z plane. Both cameras are synchronized to capture images simultaneously. Moreover, we attempted to fill in values that were missed during the recognition process. The time stamps during the detection process were used as the basis for generating the 3D trajectories.

### 2.3 Experimental environment and evaluation indicators

The recordings were conducted using a Logitech C922 Pro HD Stream Webcam, capturing videos at 60 frames/second with a resolution of 1,920 × 1,080 pixels. The aquarium, made from acrylic, measured 438 × 14 cm in length, width, and height. All deep learning processes, including training and evaluation, were performed on a computer with an i5-12500 processor, 32 GB of RAM, an NVIDIA GeForce RTX 3060, and a Windows 10 Pro x64 operating system. Data preprocessing and 3D graph plotting were done using Python 3.7. The LabelImg tool was used for dataset annotation, and network construction and training validation were conducted within a virtual environment using Anaconda3.

Three evaluation metrics were employed to assess the performance of the proposed method: accuracy (Equation 1), precision (Equation 2), and recall (also known as sensitivity or true positive rate (TPR)) (Equation 3). The variables. $T_{p}$ , $F_{p}$ , $T_{n}$ and $F_{n}$ represent the number of predicted true positives, false positives, true negatives, and false negatives, respectively. The performance metrics are defined as follows:

$$\text{Accuracy}=\frac{T_{p}+T_{n}}{T_{p}{+F}_{p}+T_{n}+F_{n}}$$ (1)

$$\text{Precision}=\frac{T_{p}}{T_{p}+F_{p}}$$ (2)

$$\text{Recall }\left(TPR\right)=\frac{T_{p}}{T_{p}+F_{n}}$$ (3)

This evaluation process not only contributes to understanding the model’s performance but also provides valuable feedback for refining the model to achieve higher accuracy and reliability. Additionally, to assess the accuracy of the identified coordinates, we utilized the Euclidean distance (Equation 4) to confirm the disparity between the recognition results and the original coordinates.

$$Euclidean\;distance=\sqrt{\left\{{\left(x_{2}-\;x_{1}\right)}^{2}+{\left(y_{2}-\;y_{1}\right)}^{2}+{\left(z_{2}-\;z_{1}\right)}^{2}\right\}}$$ (4)

## 3 Results

### 3.1 Value selection adjustment results

Here, we present the performance evaluation of the training model and the model results. First, we describe the selections used for the base model. Subsequently, we discuss the hyperparameter adjustment process, including the data methods, training iterations, and dataset size. Finally, the model performance was evaluated. Section 3.2 presents the results of the final model. Except for the initial round of weight adjustments, all tuning outcomes are validated using a separate set of 1,000 test images distinct from the training data.

**FIGURE 4:**
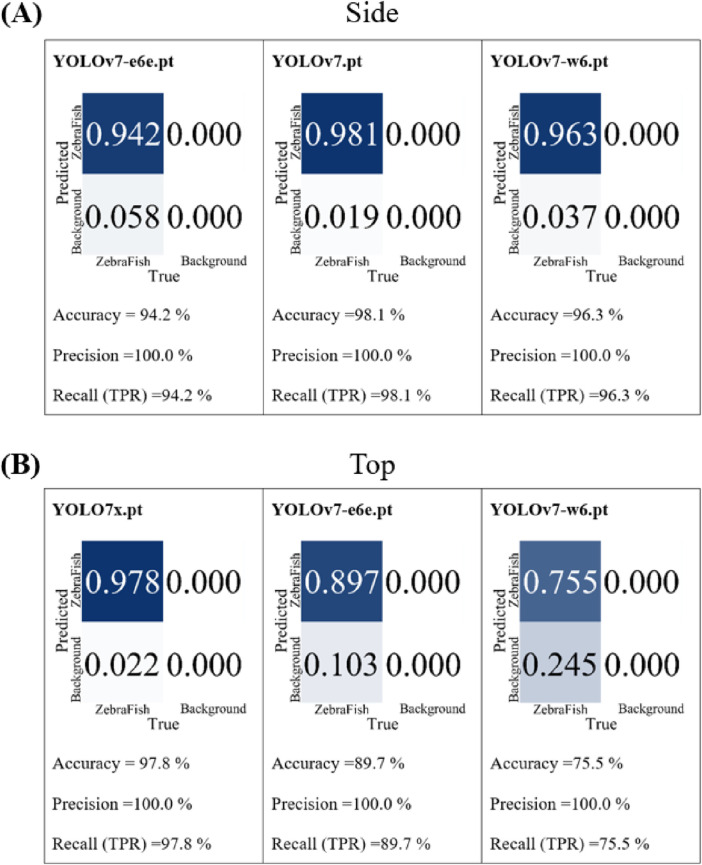
The comparison results of the basic weights for the second round of training. **(A)** For the baseline comparison of the performance of the side-view camera model, using the e6e weight, the accuracy is 94.2%, the precision is 100%, and the recall is 94.2%. With the v7 weight, the accuracy is 98.1%, the precision is 100%, and the recall is 98.1%. With the w6 weight, the accuracy is 96.3%, the precision is 100%, and the recall is 96.3%. **(B)** For the baseline comparison of the performance of the top-view camera model, using the v7x weight, the accuracy is 97.8%, precision is 100%, and recall is 97.8%. With the e6e weight, the accuracy is 89.7%, the precision is 100%, and the recall is 89.7%. With the w6 weight, the accuracy is 75.5%, the precision is 100%, and the recall is 75.5%.

YOLOv7 is equipped with six initial weights: yolo-v7.pt, yolo-e6.pt, yolo-d6.pt, yolo-e6e.pt, yolo-w6.pt, and yolo-v7x.pt. In the first round of testing, the top- and side-view camera models were separately trained 300 times with 1,000 annotated images. The results were then compared in terms of precision and recall, as these metrics are closely related to the accuracy of the detection results, with better values closer to 1. The top three performing base models were selected for the second round of testing. In the second round, 3,470 annotated images were used for individual model training (300 iterations each), and the trained models were tested with a 90% confidence threshold with the same test video. Then, the accuracy, precision, and recall of the models were compared. For the side-view camera, the precision and recall were compared in the first round, and the results in second round are shown in Figure 4A. The e6e weight exhibited an accuracy, precision, and recall of 94.2%, 100%, and 94.2%, respectively; the v7 weight had an accuracy, precision, and recall of 98.1%, 100%, and 98.1%, respectively; and the w6 weight demonstrated an accuracy, precision, and recall of 96.3%, 100%, and 96.3%, respectively. The v7 weight achieved the highest accuracy, precision, and recall; thus, the side-view camera model was trained with the yolov7.pt weight. For the top-view camera, we compared the precision and recall in the first round, and the results of the second round are illustrated in Figure 4B. The v7x weight displayed accuracy, precision, and recall of 97.8%, 100%, and 97.8%, respectively; the e6e weight exhibited an accuracy, precision, and recall of 89.7%, 100%, and 89.7%, respectively; and the w6 weight showed an accuracy, precision, and recall of 75.5%, 100%, and 75.5%, respectively. The v7x weight achieved the highest accuracy, precision, and recall; thus, the top-view camera model was trained with the yolov7x.pt weight.

**FIGURE 5:**
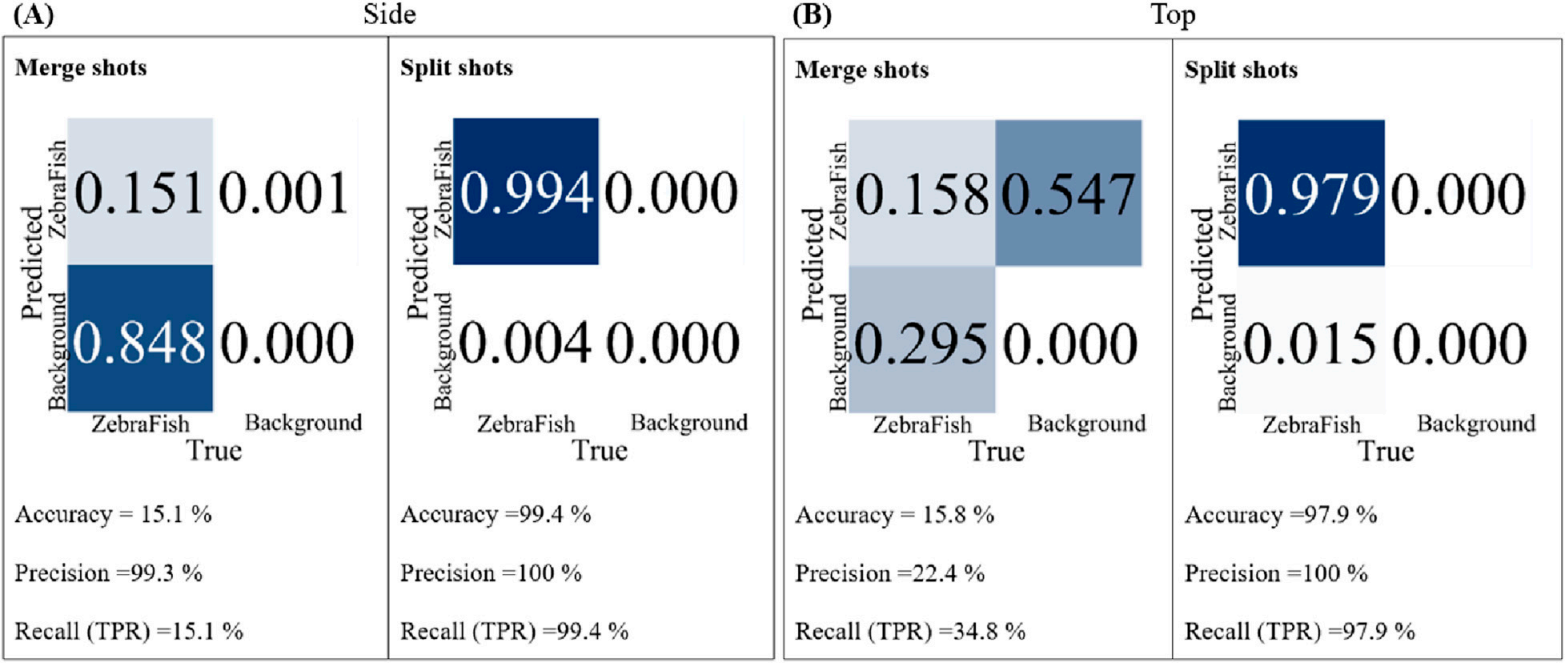
The accuracy, precision, and recall of the two models trained separately and jointly with the same test set were compared. **(A)** For the side-view camera model, for the joint training strategy, the accuracy is 15.1%, the precision is 99.3%, and the recall is 15.1%. For the separate training strategy, the accuracy is 99.4%, the precision is 100%, and the recall is 99.4%. **(B)** For the top-view camera model, for the joint training strategy, the accuracy is 15.8%, the precision is 22.4%, and the recall is 34.8%. For the separate training strategy, the accuracy is 97.9%, the precision is 100%, and the recall is 97.9%.

In this study, we utilized two cameras: top and side. Two datasets were created, each containing 1,000 images collected by two cameras. The yolov7.pt model was employed as the base model for training, and the training was conducted for 300 iterations. The trained models were subsequently tested with the same test video with a confidence threshold of 70%, and their accuracy, precision, and recall were compared. Figure 5A shows the results for the side-view camera with separate and combined training strategies. For the combined training approach, the accuracy, precision, and recall were 15.1%, 99.3%, and 15.1%, respectively. In contrast, for the separate training approach, the accuracy, precision, and recall were 99.4%, 100%, and 99.4%, respectively. Figure 5B shows the results for the top-view camera with separate and combined training strategies and different test videos. For the combined training approach, the accuracy, precision, and recall were 15.8%, 22.4%, and 34.8%, respectively. However, for the separate training approach, the accuracy, precision, and recall were 97.9%, 100%, and 97.9%, respectively. For both the top- and side-view cameras, the separately trained models exhibited superior accuracy, precision, and recall than the models trained simultaneously with data from both cameras. Therefore, subsequent tests were conducted with separately trained models for each camera.

**FIGURE 6:**
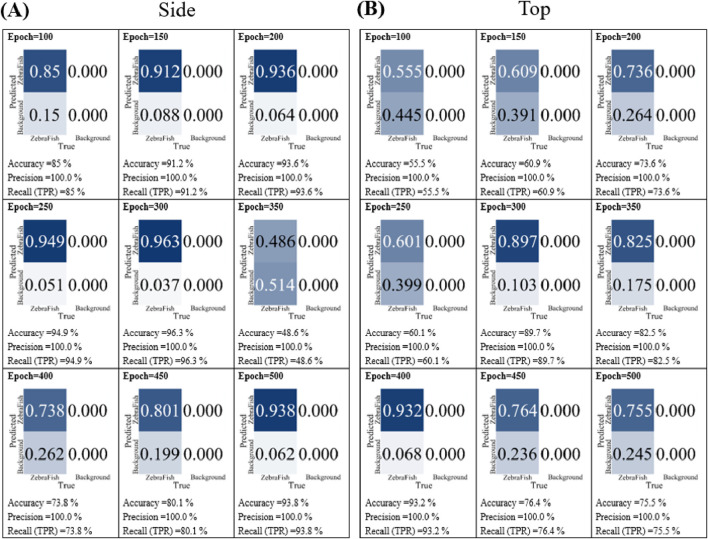
The model performance with different numbers of training iterations. **(A)** The side-view camera and **(B)** top-view camera models were trained for 100, 150, 200, 250, 300, 350, 400, 450, and 500 iterations. Subsequently, the models were separately tested with the test set, and their accuracy, precision, and recall were compared.

In the training iteration tests, the top- and side-view camera models were separately trained using the e6e and w6 weights, respectively, with 3,470 image datasets. The training was conducted for 100, 150, 200, 250, 300, 350, 400, 450, and 500 iterations. Subsequently, the models were tested with the test set, and their accuracy, precision, and recall were compared. Figure 6A shows that for 100 iterations, the accuracy, precision, and recall were 85%, 100%, and 85%, respectively. The values improved as the number of training iterations increased up to 300; however, the values rapidly decreased at 350 iterations. As the number of training iterations increased further, the accuracy and recall increased; however, even at 500 iterations, the performance did not surpass that of the model trained for 300 iterations. Figure 6B displays the results for the top-view camera using the same data with different training iterations. After 100 iterations, the accuracy, precision, and recall were 55.5%, 100%, and 55.5%, respectively. Similar to the side-view camera results, the values increased up to 200 iterations. Notably, there was a 10% decrease in accuracy after 250 iterations, and the accuracy and recall fluctuated between 80% and 96% with 350, 400, 450, and 500 iterations. In conclusion, considering the stability and high accuracy, precision, and recall values, 300 training iterations were selected for the model in this study.

**FIGURE 7:**
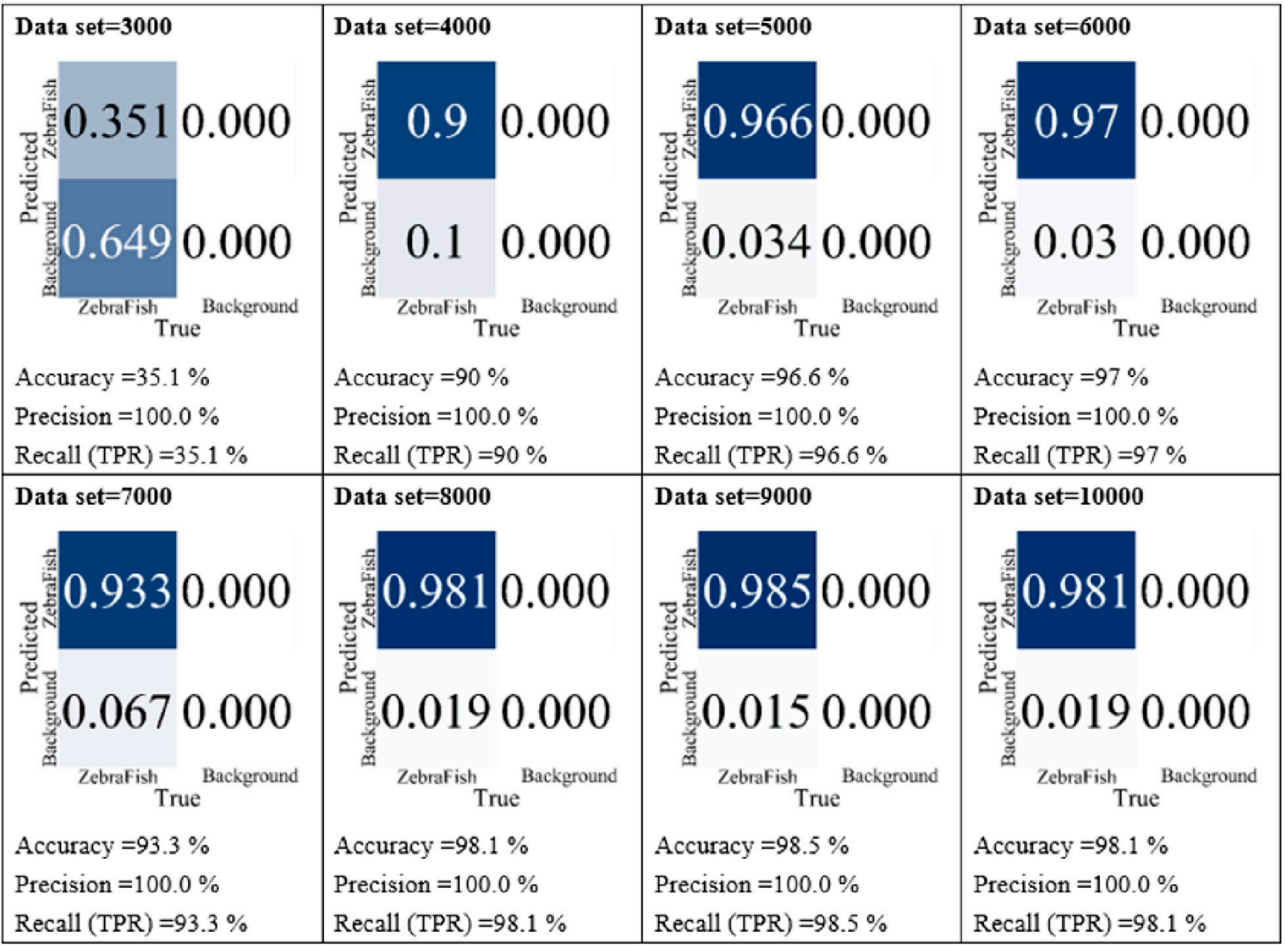
Comparison of the model performance with different datasets. Using images collected by the side-view camera as the dataset, we employed varying numbers of images, specifically 3,000, 4,000, 5,000, 6,000, 7,000, 8,000, 9,000, and 10,000 images, for model training. Subsequently, the models were individually tested with the test set, and their accuracy, precision, and recall were compared.

In the data quantity testing phase, the w6 weight was utilized for 300 training iterations, and the side-view camera images were employed as the dataset. In these experiments, datasets containing 3,000, 4,000, 5,000, 6,000, 7,000, 8,000, 9,000, and 10,000 images were used. Subsequently, the models were tested with the test set, and their accuracy, precision, and recall were compared, as shown in Figure 7. For the model trained with 3,000 images, the accuracy, precision, and recall were 35.1%, 100%, and 35.1%, respectively. As the dataset size increased, the accuracy, precision, and recall gradually increased. Notably, starting with a 4,000 image dataset, the accuracy, precision, and recall improved incrementally. For instance, the model trained with 4,000 images achieved 90% accuracy, 100% precision, and 90% recall, while the model trained with 10,000 images obtained 98.1% accuracy, 100% precision, and 98.1% recall. Based on these results, we utilized all annotated images for training. The final model training hyperparameters, dataset sizes and YOLO models are summarized in Table 2. Two camera datasets both include 18,490 images trained with YOLO-v7 for 300 iterations.

**TABLE 2:** The parameters used for final model training.

	Weight	Number of epochs	Number of images in dataset
Side-view camera	YOLO-v7	300	18,490
Top-viewcamera	YOLO-v7x	300	18,490

The side-view camera model dataset consists of a total of 18,490 images, and training was conducted using YOLO-v7 for 300 iterations. Additionally, the side-view camera model dataset includes 18,490 images, and the model was trained with YOLO-v7x for 300 iterations.

### 3.2 Parameter evaluation

**FIGURE 8:**
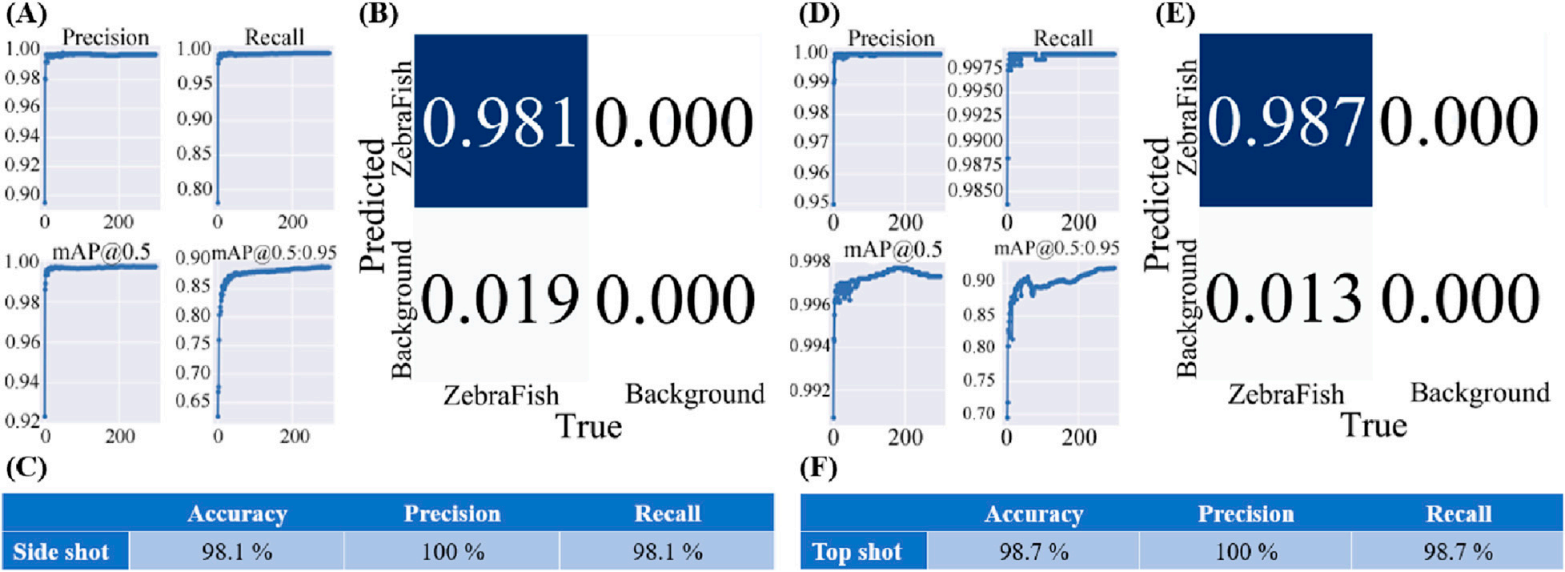
Training results of the final model used in this study. **(A)** The training results for the side-view camera model, with the accuracy approaching 100%, recall approaching 100%, and average mAP accuracy approaching 100% for each training iteration with an IoU threshold greater than 0.5. Additionally, the average mAP accuracy for IoU thresholds ranging from 0.5 to 0.95 approaches 100%. **(B)** The confusion matrix generated after applying the model to the validation set. **(C)** The accuracy of the side-view camera model is 98.1%, the precision is 100%, and the recall is 98.1%. **(D)** The training results of the top-view camera model, showing an accuracy approaching 100%, a recall approaching 99.75%, and an average mAP accuracy approaching 99.8% for each training iteration with an IoU threshold greater than 0.5. The average mAP accuracy for IoU thresholds ranging from 0.5 to 0.95 approaches 98%. **(E)** The confusion matrix generated after applying the model to the validation set. **(F)** The accuracy of the top-view camera model is 98.1%, the precision is 100%, and the recall is 98.1%.

Figure 8A shows the model training results, while Figure 8B displays the confusion matrix generated by applying the model to the validation set, illustrating the model’s detection results for the training set. The model accuracy was 98.1%, and the precision and recall both reached 100% (Figure 8C). Similarly, Figure 8D shows the model training results, and Figure 8E presents the confusion matrix generated by applying the model to the validation set. The model achieved an accuracy of 98.1%, with a precision and recall of 100% (Figure 8F).

Additionally, the detection results of the final model and the initial model with the test set are presented in Table 3. The test set includes 3,632 coordinate points. The final model successfully detects all 3,632 points, with the error ranging from 0.006 cm to 0.284 cm when compared to the ground truth of the test set. The average error was 0.428 cm. In contrast, the initial model detects only 1,329 points, exhibiting the error ranging from 0.044 cm to 10.892 cm compared to the ground truth of the test set, with an average error of 4.46 cm. Compared to the initial model, the final model detects 173.11% more coordinates. Furthermore, compared to the ground truth of the test set, the final model reduces the error range by 86.36%–97.39%, with a significant 90.5% decrease in the average error.

**TABLE 3:** Performance comparison of the initial and final models.

value	Coordinates	Maximum difference	Minimum difference	Average difference
Sources and comparisons				
Test data	3,632	—	—	—
Initial model	1,329	10.892 cm	0.044 cm	4.467 cm
Final model	3,632	0.284 cm	0.006 cm	0.428 cm
Difference	+173.11%	−97.39%	−86.36%	−90.5%

Based on the test set with 3,632 coordinates, the initial model detected 1,329 coordinates, with errors ranging from 0.044 cm to 10.892 cm. The average error was 4.46 cm. The final model, on the other hand, detected all 3,632 coordinates, with errors ranging from 0.006 cm to 0.284 cm. The average error for the final model was 0.428 cm. Compared to the initial model, the final model identified 173.11% more coordinates. The error range decreased by 86.36%–97.39%, and the average error was reduced by 90.5%.

### 3.3 Trajectory reproduction results

**FIGURE 9:**
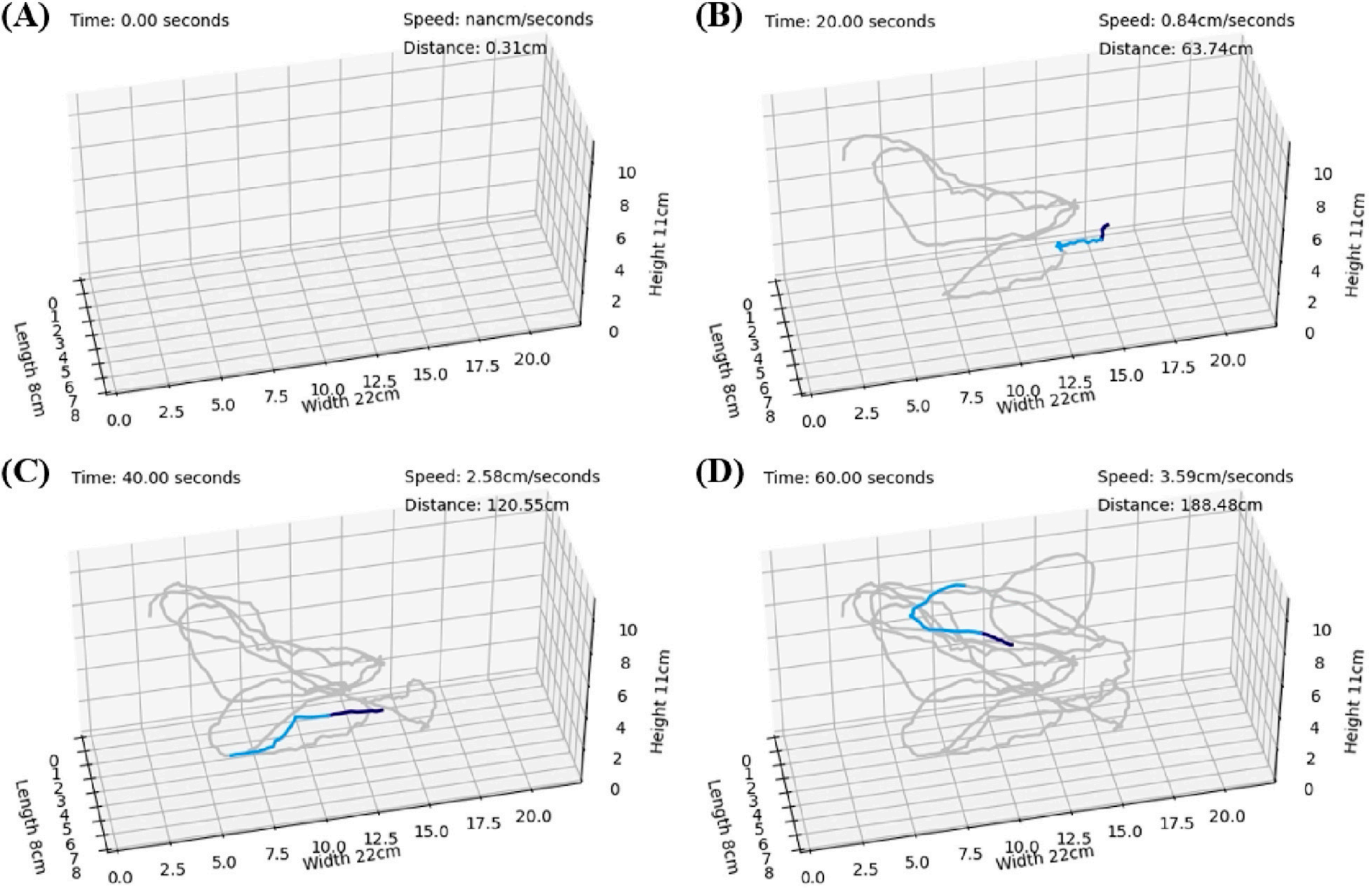
The trajectories connected on the 3-axis coordinates representing the restored aquarium dimensions. The trajectories for the most recent 1 s are depicted in deep blue, those for the most recent 2–3 s are shown in light blue, and the remaining trajectories are shown in gray. In the upper right corner, the cumulative distance traveled and the instantaneous velocity are presented. **(A)** Represents the state at 0 s. **(B)** Represents the state at 20 s. **(C)** Represents the state at 40 s. **(D)** Represents the state at 60 s.

After model training, the detection results were organized and merged to reconstruct the dynamic 3D trajectories. To illustrate the dynamic paths, we present the results obtained by capturing images at intervals of 20 s from 0.0 to 60.0 s. Figures 9A–D show the results at 0, 20, 40, and 60 s, respectively. In these figures, the trajectory of the latest 1 s is represented in deep blue, the trajectory of the latest 2–3 s is shown in light blue, and the remaining trajectories are depicted in gray.

## 4 Discussion

Since its development in 2015, the YOLO series of models has been consistently updated, with nearly one new version introduced each year. The v7 version used in this study was proposed by Wang et al. (2022) in July 2022. Although a new model, YOLOv6, was released by the Chinese e-commerce group Meituan in June 2022 (Chuyi Li et al., 2022), it was not officially included in the version comparison due to the lack of a fair comparison. Therefore, researchers continued to apply YOLOv5 before the release of YOLOv7. YOLOv5, released in June 2020 (ultralytics, 2020), gained popularity due to its simple design and relatively lightweight model structure, achieving a balance between speed and accuracy that satisfied many users. YOLOv5 became the preferred model for real-time object detection in most engineering applications. Two years later, YOLOv7 was introduced, which substantially improved the model architecture, resulting in faster recognition and increased accuracy. The changes in the convolution process provided more gradient diversity for different feature maps, reducing disruptions in the residual connections of ResNet and the connections in DenseNet.

We chose YOLOv7 as the primary model for this study based on our target characteristics. For zebrafish, which are small with fast movements, precise recognition is preferred over a lightweight model. This precision is crucial for obtaining accurate coordinates for trajectory reconstruction. Additionally, zebrafish movements in water present challenging scenarios for manual recognition, demanding more intricate and sophisticated detection methods.

The application of YOLOv7 in zebrafish tracking is both innovative and beneficial compared to previous methods. YOLOv7 offers improved detection accuracy and faster processing speeds, enabling quick and precise handling of many high-resolution images. This feature is well suited for specific application needs in zebrafish monitoring. By leveraging YOLOv7, we achieve significant advancements in both accuracy and efficiency, marking a substantial innovation in the field of aquatic organism tracking. This approach provides a new level of precision and speed, which were previously unattainable with traditional methods.

Moreover, by selecting an appropriate model and dataset, computer vision approaches can address scenarios that cannot be recognized by the human eye. To optimize model performance, we conducted a series of tests to evaluate the model parameters to ensure their effectiveness.

In the selection of the initial YOLOv7 model, we trained all the weights and then chose the optimal weights based on the model performance with the test set. Under the same training conditions, we compared the accuracy, recall, and precision of the models with the validation set. In the second round of training for the side-view camera, all three weights (e6, v7, w6) achieved a minimum of 94.2% accuracy, 100% recall, and 94.2% precision (Figure 4A), indicating that YOLOv7 is highly suitable for this research.

The training data for this study were collected simultaneously by two cameras. The images collected by both cameras were merged, annotated, and using for training a single model. While the model could learn features from both perspectives concurrently, mutual interference led to decreased model accuracy. We observed this in our research, as the accuracies of the top-view and side-view camera models were only 15.8% and 15.1%, respectively (Figure 5A). An alternative approach involved annotating the images collected by each camera separately, resulting in distinct models for the top- and side-view cameras. This method allowed the model to focus on learning features from each individual camera, thereby enhancing accuracy. Our tests validated this finding, with training accuracies of 97.9% (top-view camera) and 99.4% (side-view camera) (Figures 5A, B).

In general, an increase in the number of training cycles provides the model with more opportunities to learn the training data rather than general features. However, this may lead to overfitting, where the model performs well with the training data but poorly with test data. The potential for overfitting increases as the number of training epochs increases, resulting in decreased model generalizability. In our study, the model demonstrated optimal performance after 300 training cycles (accuracy of 96.3%) (Figure 6A). Further increasing the number of cycles tended to lead to overfitting, as the model trained for 500 cycles exhibited an accuracy of only 75.5% (Figure 6B). This indicates that additional training cycles did not improve the model’s performance with the validation set compared to that of the model trained for 300 cycles.

Training deep learning models requires substantial sample data. Increasing data diversity enhances the model’s generalizability, reduces overfitting, and improves handling of new, unseen data. More data also stabilizes the training process, smoothing gradient descent, and improving model performance and accuracy. In our study, experiments with different dataset sizes revealed that the model accuracy increased with increasing amount of data (Figure 7), indicating that the model learned more patterns and features when larger datasets were used.

By comparing different test models, we can identify suitable hyperparameters for target recognition, significantly enhancing results (Table 3). We compared the performance of the initial and final models using a test set of 3,632 coordinates. The final model recognized 173.11% more coordinates than the initial model. Compared to the ground truth, the final model’s maximum, minimum, and average errors were reduced by 97.39%, 86.36%, and 90.5%, respectively. These results demonstrate that the model, after parameter adjustments, has greatly improved accuracy and generalizability.

**FIGURE 10:**
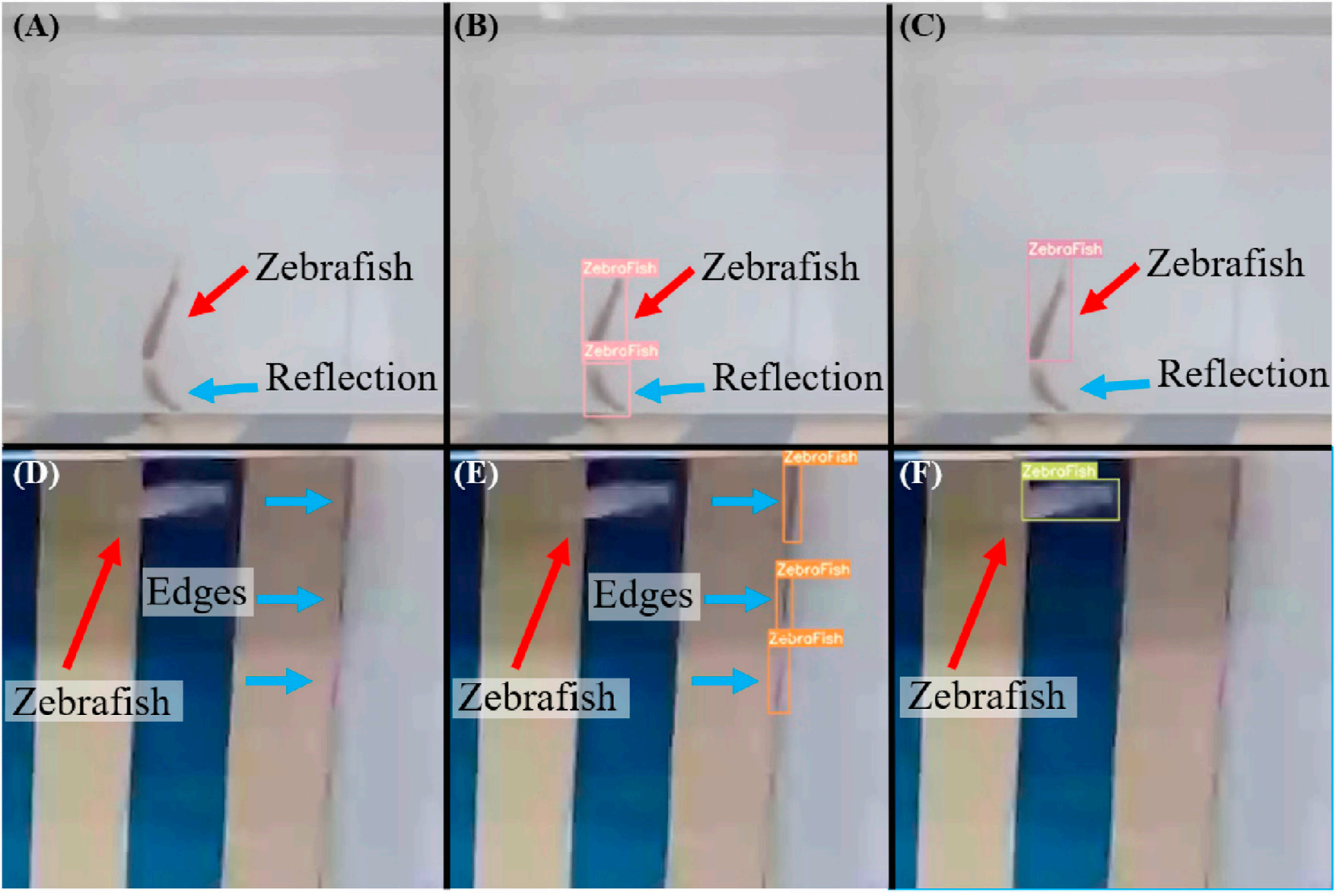
Common situations leading to potential misidentification of zebrafish. **(A)** Zebrafish swimming at the edge of an aquarium, which often leads to reflections that can reduce detection performance. **(B)** A poorly performing model may misinterpret reflections as actual zebrafish, leading to erroneous results. **(C)** Through iterative experiments with different initial weights, numbers of training iterations, and dataset sizes as hyperparameters, the model’s performance is assessed to determine the most suitable parameters for training. The performance of the proposed model was subsequently validated using a test set to confirm the performance with the chosen parameters, ensuring that the trained model accurately identified the zebrafish. **(D)** Zebrafish appearing blurry in images when moving at high speeds. **(E)** Without using appropriate parameters to train the model, significant deviations from the original object may occur. **(F)** A model trained after parameter adjustments can successfully identify zebrafish even when they are moving at high speeds.

As shown in Figure 10A, when zebrafish swim to the edge of the fish tank, reflections can occur, which may lead to challenging detection scenarios. Inefficient models may misinterpret the reflection as zebrafish (Figure 10B), leading to erroneous results. Through iterative experiments with initial weights, training cycles, and data volumes as hyperparameters, the model’s performance was assessed to determine the most suitable parameters for training. The model was subsequently validated using a test set to evaluate its accuracy, precision, and recall, ensuring the goodness of fit of the selected parameters. The trained model accurately identified the correct zebrafish (Figure 10C). Furthermore, in situations where zebrafish move rapidly, they appear blurred in images (Figure 10D). Without appropriate parameter tuning during model training, significant deviations from the original object may occur. For instance, edge residues can be misinterpreted as zebrafish while failing to recognize the actual zebrafish (Figure 10E). However, by adjusting hyperparameters such as the number of training cycles, data volume, data type, and weights, the model successfully identified zebrafish even in high-speed motion scenarios (Figure 10F).

In this study, we successfully utilized target position identification and timestamp approaches to reconstruct the 3D movement trajectories of zebrafish, demonstrating the potential of deep learning in automating zebrafish trajectory tracking. This method allows accurate tracing of zebrafish motion trajectories, providing valuable data and insights. This approach can enhance our understanding of zebrafish behavior and ecological habits, crucial in both laboratory and field research. This method has broad applicability (Liu et al., 2023; Wu et al., 2023), improving our understanding of neuroscience and behavior, and contributing to drug development, environmental toxicity assessment, genetic research, and brain-computer interface technology. Zebrafish’s simple nervous system makes them ideal for analyzing neural networks and related behaviors, aiding in understanding human neurological diseases. Rapid screening and evaluation of drugs through trajectory tracking improve drug development efficiency and reduce the need for animal experiments. This method is also useful for assessing environmental toxins’ impact on ecosystems, promoting environmental protection and risk assessment. The genetic similarity between zebrafish and humans provides valuable information for genetic research and supports brain-computer interface technology development, potentially aiding neurological disease treatments.

We acknowledge the limitation of using a single zebrafish in our study, which may affect the generalizability of our results. This constraint could potentially impact the robustness and applicability of our findings across different contexts. To address this issue, we suggest future research to expand the dataset to include a broader range of subjects. This would enhance the validity of the conclusions and provide more comprehensive insights into the studied phenomena.

At the same time we believe it is important to extend this approach to accommodate multiple subjects simultaneously. To address this, Future research should build upon the existing methods to develop additional identification techniques that address more complex computational issues, such as resolving overlapping trajectories and multi-object tracking. We propose exploring advanced tracking techniques and enhancing our dataset to support multi-object tracking in future research. Overall, this research is highly important for promoting the advancement of medical and environmental science, as well as improving human health and quality of life.

## 5 Conclusion

3D trajectory tracking for zebrafish is crucial in the field of biomedical research. In zebrafish studies, the motion trajectories, behaviors, and physiological responses of zebrafish must be determined to understand the mechanisms of diseases such as neurological disorders, heart diseases, and cancer. Compared to 3D trajectory tracking methods, traditional manual observation and 2D trajectory tracking techniques are insufficient for accurate motion trajectory and behavioral analyses for zebrafish. This limitation can potentially lead to inaccurate results in experiments. This study introduces a novel approach for 3D zebrafish trajectory tracking utilizing a dataset of 36,980 images to reconstruct the coordinates of 18,490 zebrafish. Through iterative comparison experiments, optimal weight hyperparameters were determined, achieving an accuracy, precision, and recall of 98.1%, 100%, and 98.1%, respectively. The highest accuracy, precision, and recall during training iteration comparisons were 96.3%, 100%, and 96.3%, respectively. During dataset size testing, the highest accuracy, precision, and recall were 98.5%, 100%, and 98.5%, respectively.

The final model for the side-view camera, trained 300 times using the v7 weight, achieved 98.1% accuracy, 100% precision, and 98.1% recall. The final top-view camera model, trained with the v7x weight, achieved 98.7% accuracy, 100% precision, and 98.7% recall. With respect to the test set including 3,632 3D coordinates, the final model identified 173.11% more coordinates than the initial model. When calculating the error between the identified and ground truth coordinates in the test set, compared with that of the initial model, the error range of the final model was reduced by 86.36%–97.39%, with the average error reduced by 90.5%. Future applications of this method for various aspects of zebrafish research, such as speed comparisons, hotspot detection, and related behavioral patterns expressed through movement, are anticipated. This approach will enable researchers to better understand zebrafish behaviors and physiological responses, thereby enhancing experimental efficiency and research quality. Overall, the proposed zebrafish trajectory tracking method is a powerful tool for in-depth exploration of behavior, neuroscience and disease, with positive impacts on innovation and development in related fields.

## Data Availability

The raw data supporting the conclusions of this article will be made available by the authors, without undue reservation.
